# A105 CHRONIC DIARRHOEA AND ELEVATED LIVER ENZYMES IN A PATIENT WITH AUTOIMMUNE POLYGLANDULAR SYNDROME TYPE 1

**DOI:** 10.1093/jcag/gwac036.105

**Published:** 2023-03-07

**Authors:** T Yodoshi, D G Valdes, I Siddiqui, B Ngan, A Muise, M J Gould

## Abstract

**Background:**

Autoimmune polyendocrine syndrome type 1 (APS1) is a rare inherited autoimmune disease, characterized by primary adrenocortical insufficiency and hypoparathyroidism. Pathologic mutations in the autoimmune regulator (AIRE) gene, which participates in the regulation of T-cell self-tolerance can lead to APS-1. Defective function of AIRE encourages production of multiple anti-cytokine and organ-specific autoantibodies, leading to severe autoimmune disease across multiple organs. Autoimmune enteropathy is one of the autoimmune conditions associated with APS-1. Symptoms include chronic diarrhoea and weight loss. Extraintestinal manifestations can include haemolytic anaemia and hepatitis.

**Purpose:**

To report on a patient with APS-1 who exhibited exacerbations of acute-on-chronic diarrhoea and transaminitis.

**Method:**

We describe the case of a 14-year-old female, the first child of healthy, first-cousin consanguineous parents, with a history of APS type 1 with a homozygous disease-causing variant in the AIRE gene (c.415C>T, p.139*), hypoparathyroidism and Addison’s disease who presented with worsening diarrhoea and unintentional weight loss (15kg) in the past year. Upper and lower endoscopies performed 10 months and 2 months prior to this admission revealed chronic gastritis and patchy active colitis, without features of a specific etiology. Enteroendocrine cells were present. Celiac disease, inflammatory bowel disease, eosinophilic gastroenteritis, and IPEX syndrome were clinically and histologically ruled out.

**Result(s):**

On admission, the patient was having five watery stools per day with associated hypocalcemia and hypokalemia which were felt to be more resistant to treatment than should be explained by her Addison’s disease alone. Albumin was preserved (46 g/L). A stool PCR test for Adenovirus was positive, though serum adenovirus PCR was negative. ALT was 231 U/L and AST was 283 U/L. IgG level was elevated (17.7 g/L), anti-nuclear antibody (ANA) was positive (1:320), and anti-smooth muscle antibody (ASMA) was also positive (1:160). It was therefore suspected that he had autoimmune enteropathy and autoimmune hepatitis, and a liver biopsy was performed as well as upper and lower endoscopies. Liver biopsy demonstrated only minimal macrosteatosis (less than 5%) but no evidence of hepatitis. No abnormal endoscopic findings were observed. Histology showed no typical features of autoimmune enteropathy. Enteroendocrine cells were noted in both small and large intestinal biopsies. Her elevated liver enzymes and diarrhea were resolved spontaneously over a 2-week period.

**Conclusion(s):**

We present a patient with APS-1 with acute-on-chronic diarrhoea and transaminitis with elevated IgG and positive ANA and ASMA. Endoscopy and liver biopsy did not reveal evidence of autoimmune enteropathy or autoimmune hepatitis. This case raises the importance of maintaining a broad differential diagnosis when diarrhoea is encountered in APS-1 as this may occur for reasons outside of autoimmune enteroendocrine cell loss.

**Please acknowledge all funding agencies by checking the applicable boxes below:**

None

**Disclosure of Interest:**

None Declared

ENDOSCOPY, TECHNOLOGY & IMAGING

